# TLC Screening for Antioxidant Activity of Extracts from Fifteen Bamboo Species and Identification of Antioxidant Flavone Glycosides from Leaves of *Bambusa. textilis* McClure

**DOI:** 10.3390/molecules171012297

**Published:** 2012-10-19

**Authors:** Jin Wang, Yong-De Yue, Feng Tang, Jia Sun

**Affiliations:** SFA Key Laboratory of Bamboo and Rattan Science and Technology, International Centre for Bamboo and Rattan, No.8 Futong Dongdajie, Wangjing, Chaoyang District, Beijing 100102, China; Email: wangjin@icbr.ac.cn (J.W.); fengtang@icbr.ac.cn (F.T.); sunjia@icbr.ac.cn (J.S.)

**Keywords:** bamboo leaves, *Bambusa. textilis* McClure, TLC bioautography, DPPH, antioxidative activity, structure elucidation, flavone *C*-glycosides

## Abstract

Interest in the antioxidant activity of bamboo leaves is growing. To discover new sources of natural antioxidants, a TLC bioautography method combined with a new image processing method was developed to evaluate the antioxidant activity of leaf extracts from 15 different species of bamboo. The results showed that the methanolic extract of *Bambusa. textilis* McClure possessed the highest antioxidant activity among the selected bamboo species. To rapidly identify the antioxidant compounds, the crude extract of *B. textilis* McClure was analysed by HPLC-UV, and HPLC-micro-fractionation of the extract was carried out. Based on TLC bioautography-guided fractionation, three antioxidant fractions were isolated from *B. textilis* McClure by preparative chromatography. These three antioxidant compounds were identified as isoorientin 4''-*O*-β-D-xylopyranoside (**1**), isoorientin 2''-*O*-α-L-rhamnoside (**2**) and isoorientin (**3**) according to their UV, MS, and NMR data. The proposed TLC screening method could therefore be an easy way to evaluate the antioxidant activity of bamboo leaves, and the results achieved should prove very helpful for promoting their utilization, as *B. textilis* McClure can be considered a promising plant source of natural antioxidants.

## 1. Introduction

There are more than 1250 bamboo (Graminaceae) species distributed all over the World. Bamboo leaves have been used clinically in some Asian countries in the treatment of fever and hypertension [1,2]. The medicinal effects of bamboo leaves are mostly attributed to their antioxidant capacity [3]. In addition to its use in traditional medicine, an extract from the leaves of *P. nigra* var. *henonis* (antioxidant of bamboo leaves, AOB) is used as a food antioxidant in China [4]. Some functional components from bamboo leaves, such as flavonoids, lactones and phenolic acids were reported [5]. Antioxidants are a group of compounds which possess the ability to protect the body from damages caused by free radicals. Several studies have been carried out to identify the antioxidants from different species of bamboo, such as *Phyllostachys edulis* [1], *Fargesia robusta* [3], and *Sasa borealis* [6]. Antioxidant chlorogenic acid derivatives and flavonoids have been identified from the leaves of *P. edulis* and *F. robusta*, respectively [1,3]. Thus, the antioxidative compounds from different species of bamboo could be different. Moreover, the antioxidative components in bamboo leaves are not fully known. Therefore, some strategies have to be designed for the screening and identification of antioxidative compounds from bamboo leaves. 

A great number of TLC techniques have been developed and successfully applied for qualitative and quantitative analysis of antioxidants [7,8], and the stable free radical 2,2-diphenyl-1-picrylhydrazyl (DPPH) was often used as a derivatization reagent for this purpose [9]. In the screening of antioxidants, the TLC bioautography assay is the method of choice due to several advantages that include flexibility, simplicity and high throughput [10,11,12]. *B. textilis* McClure is a kind of bamboo species used in traditional medicine in China. A metabolic study previously performed by our group reported that three bioactive compounds from *B. textilis* McClure were identified [13]. To the best of our knowledge, the active antioxidant compounds of *B. textilis* McClure have not been identified. 

In this study, we evaluated the antioxidant activities of 15 different species of bamboo using a TLC bioautography assay. The methanolic extract of *Bambusa. textilis* McClure showed the strongest antioxidant activity among the selected bamboo species. The main antioxidant compounds of *Bambusa. textilis* McClure were isolated and identified based on preparative-HPLC, MS, and NMR techniques.

## 2. Results and Discussion

### 2.1. TLC Bioautography Assay

**Figure 1:**
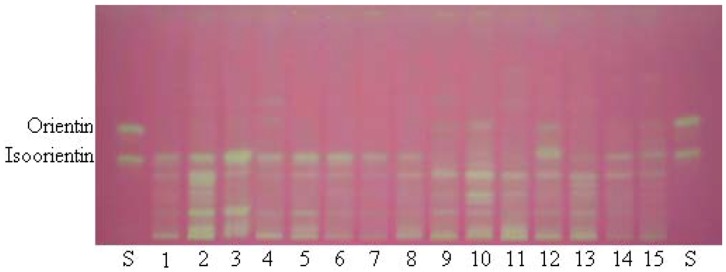
Typical TLC photography of standards and extracts from the different species of bamboo colorized with 0.05% DPPH. S: Standards are isoorientin and orientin. The different bamboo species (No. 1–15) are listed in Table 1.

To screen the antioxidant capacity of bamboo leaves, a TLC bioautography method was performed. After separation on TLC plates, the compounds with radical scavenging activity were determined *in situ* with DPPH reagent. The TLC plate was observed with visible light (Figure 1).

As shown in Figure 1, the samples producing yellowish bands on the red background were considered as antioxidants. Usually, the purple background colour was visualized after spraying the plate with DPPH reagent [14,15]. The background color of the plate changed from purple to red after 12 h in darkness. The red background makes the yellowish bands clearly visible, which benefits the next image processing.

**Table 1:** Investigated leaves of 15 different species of bamboo.

No.	Family	Plant scientific name	Sampling Location	
1	*Bambusa*	*B. multiplex* cv. *Alphonse-Karr*	Nanjing, China
2	*Bambusa*	*B. textilis* McClure	Nanchang, Jiangxi Province, China
3	*Bambusa*	*B. multiplex* cv. *Silverstripe*	Nanchang, Jiangxi Province, China
4	*Bambusa*	*B. ventricosa* McClure	Nanchang, Jiangxi Province, China
5	*Bambusa*	*B. multiplex* var. *multiplex*	Nanjing, China
6	*Bambusa*	*B. multiplex* cv. *Fernleaf*	Nanchang, Jiangxi Province, China
7	*Bambusa*	*B. multiplex* var. *riviereorum* R.Maire	Nanchang, Jiangxi Province, China
8	*Bambusa*	*B. pervariabilis* McClure	Nanning, Guangxi Province, China
9	*Bashania*	*B. fargesii* (E.G. Camus) Keng f.et Yi	Nanjing, China
10	*Brachystachyurn*	*B. densiflorum* (Rendle) Keng	Nanchang, Jiangxi Province, China
11	*Chimonobambusa*	*Ch. quadrangularis* (Fenzi) Makino	Nanchang, Jiangxi Province, China
12	*Chimonocalamus*	*Chi. delicatus* Hsueh et Yi	Jinping, Yunnan Province, China
13	*Dendrocalamus*	*D. oldhamic* McClure	Nanchang, Jiangxi Province, China
14	*Dendrocalamus*	*D. minor* var*. amoenus* (Q.H.Dai et C.F.Huang) Hsueh et D.Z.Li	Yibin, Sichuan Province, China
15	*Fargesia*	*F. hsuehiana* Yi	Jinping, Yunnan Province, China

### 2.2. Image Processing

**Figure 2:**
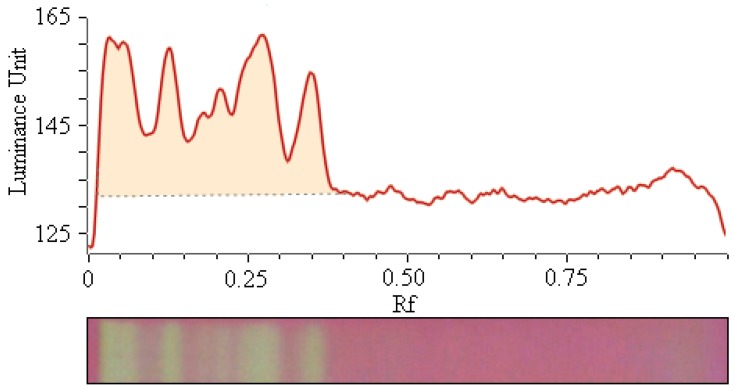
An example of TLC chromatogram and integration of peaks from leaf extracts of *B. textilis* McClure.

After separation of the antioxidant compounds of the bamboo-leaf extracts using thin-layer chromatography (TLC), the derivatization was carried out by the DPPH reagent. The TLC photos were examined with ChemPattern 2 Professional Version (a kind of chromatography fingerprint processing software). Finally, the yellowish bands on the TLC plates were converted to the corresponding chromatographic peaks. The chromatographic response (peak area) is directly related to the antioxidant ability of the extract [16]. Hence, total peak area of an extract can be considered as the sum of antioxidant activities of all ingredients from the extract. The aim of image processing is to obtain the total peak area. The baseline of peaks was marked manually as one line from the start to the end (Figure 2).

### 2.3. Comparison of Antioxidant Activity of Selected Bamboo Leaves

**Figure 3:**
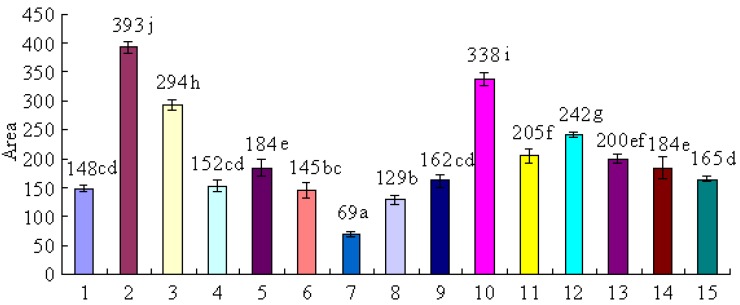
Comparison of antioxidant capacities of fifteen species of bamboo. Each value is the mean of triplicate measurements ± standard deviation. Different letters (a–j) on the top of the columns were significantly different (*p* < 0.05). Different bamboo species (No. 1–15) are listed in Table 1. For the detailed protocol for evaluation of antioxidant activity see the Experimental section.

The antioxidant activity of bamboo leaves can be compared on the basis of their total peak areas. By statistical analysis, different antioxidant activities of the extracts from 15 species of bamboo leaves are shown in Figure 3. 

As shown in Figure 3, among the selected bamboo species, the extract of *B. textilis* McClure (No. 2) exhibited the highest antioxidant activity. To better analyse its antioxidant activity contribution, we next needed to further isolate and identify the main antioxidant compounds of *B. textilis* McClure.

### 2.4. Screening and Isolation of Antioxidant Compounds

**Figure 4:**
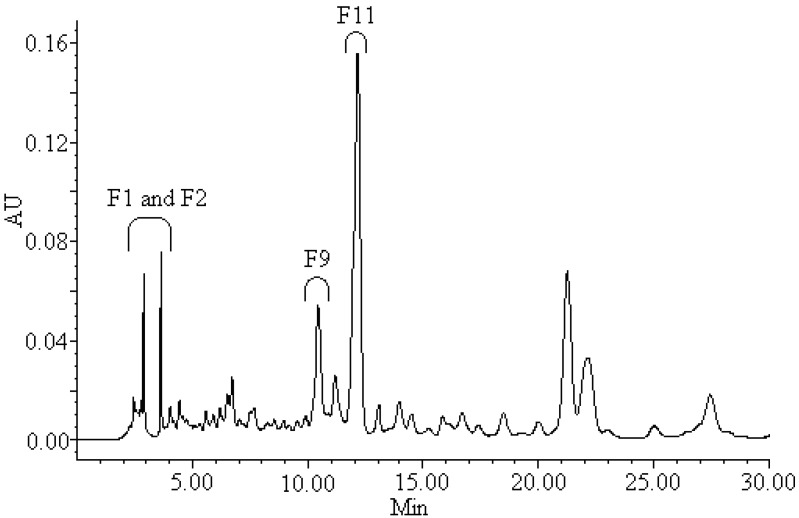
HPLC chromatogram (330 nm) of the aqueous methanolic extract of *B. textilis* McClure with indication of the active antiradical fractions. Fractions (F1, F2, F9 and F11) of the extract presented antioxidant activity.

To identify the main antioxidant compounds, the leaf extract of *B. textilis* McClure was fractionated by HPLC. The HPLC chromatogram is shown in Figure 4.

**Figure 5:**
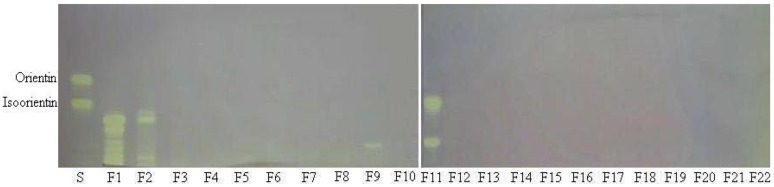
TLC photography of standards and fractions of *B. textilis* McClure leaf extracts colorized with 0.05% DPPH. S: Standards are isoorientin and orientin. Lanes F1–F22 in the picture correspond to fraction F1 to fraction F22, respectively.

Each fraction of the crude extract was evaluated for its antioxidant capacity using the TLC bioautography assay. Antioxidant compounds were visualized as yellow spots on the TLC plates. The TLC bioautography profile of different fractions indicated that only four fractions (F1, F2, F9 and F11) showed obviously yellow spots against the purple background (Figure 5).

As seen from Figure 5, TLC analysis of the fractions F1 and F2 showed a series of yellow bands. On the basis of their UV spectra, the compounds present in fractions F1 and F2 were mainly phenolic acids. It can be found from Figure 4 and Figure 5 that the fractions F1 and F2 contained too many compounds, and each compound was less abundant, thus, the fractions F1 and F2 were not considered further in this study. Fraction F9 showed an single active component (compound **1**), while TLC revealed that fraction F11 was still not pure and contained two components. Based on preparative HPLC, fraction F11 was separated into two sub-fractions: F11-1 and F11-2 (corresponding to compounds **2** and **3**). Thus a total of three antioxidant compounds (compounds **1**, **2** and **3**) were obtained from the crude extract of *B. textilis* McClure by preparative chromatography.

### 2.5. Structure Identification of the Three Antioxidant Compounds

**Figure 6:**
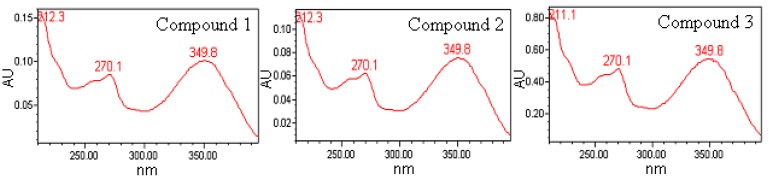
UV spectra of the three antioxidant compounds.

The UV spectra of three antioxidant compounds are shown in Figure 6. 

The typical UV spectra showed that three antioxidant compounds belong to flavonoids according to their two major absorption peaks at 250–360 nm. The chemical structures of flavonoids are based on a C15 skeleton with two aromatic rings (A and B) and one heterocycle ring C [17]. Absorption at 349.8 nm suggested that the compounds belong to the flavone family unsubstituted at 3-position. Peaks (270.1 nm) with shoulders indicated the presence of hydroxyl groups in the B ring.

### 2.6. LC-Q-TOF-MS Analysis

**Figure 7:**
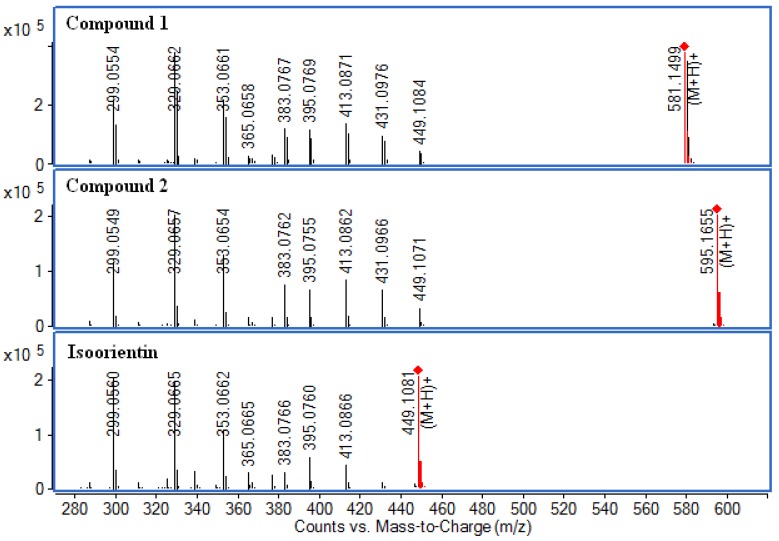
(+)ESI-MS/MS mass spectra of compounds **1**, **2** and isoorientin.

In the LC-Q-TOF-MS analysis, the precursor ions and fragment ions from three compounds were observed. Accurate mass data and isotopic distributions for the precursor and product ions were studied and compared to spectral data of reference compound (Figure 7).

As seen from Figure 7, three molecular ions at *m/z* 581.1499 [M+H]^+^, *m/z* 595.1655 [M+H]^+ ^and *m/z* 449.1081 [M+H]^+^ were observed. The main MS fragments of compound **1** and **2** were the same as those of an isoorientin standard. Among these fragment ions, the major ions for both compound **1** and **2** were at *m/z* 299.0554 [M+H−150]^+^ and *m/z* 329.0662 [M+H−120]^+^. In addition, losses of some neutral molecules such as H_2_O (18 Da) suggested flavonoids with two hydroxy (-OH) groups in *ortho* positions. 

As for compound **1**, a fragment at *m*/*z* 449.1084 [M+H−132]^+^, corresponded to loss of one pentose unit (132 Da), with a low-intensity and another fragment at *m*/*z* 431.0976 [M+H−132−H_2_O]^+^ with higher intensity, as well as the absence of the aglycone ion is in agreement with an *O*,*C*-diglycoside structure. The relative intensity above 90% of the sugar unit is characteristic of di-*O*,*C*-glycosides [18]. Thus, compound **1** could have a pentose unit in the terminal position of a disaccharide unit. The presence of the ion at *m*/*z* 329.0662 [M+H-−132−120]^+^ and the absence of a [M+H−132−60]^+^ fragment indicated a hexose as the *C*-glycosylation sugar. 

In addition, compound **2** presented a weak ion at *m*/*z* 449.1071 [M+H−146]^+^ corresponding to the loss of one deoxyhexose unit (146 Da). A major ion at *m*/*z* 431.0966 [M+H−146−H_2_O]^+^ was also observed. The MS/MS fragmentation pattern of compound **2** was typical of *O*,*C*-diglycosides. Compound **2** could have a deoxyhexose unit in the terminal position of a disaccharide unit. The presence of the ion at *m*/*z* 329.0657 [M+H−146−120]^+^ and the absence of the [M+H−146−60]^+^ fragment indicated a hexose as the *C*-glycosylation sugar. Compound **3** was identified by comparing its UV spectrum, mass data, and retention time with those of an isoorientin standard. 

### 2.7. Acid Hydrolysis of Compound ***1*** and ***2***

**Figure 8:**
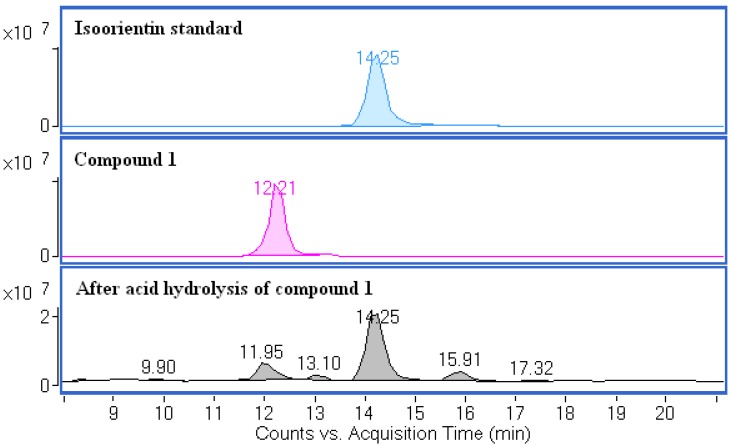
(+)ESI-Q-TOF-MS total ion chromatograms (TIC) of an isoorientin standard, compound **1**, and degradation products of compound **1** after acid hydrolysis.

According to the LC-Q-TOF-MS analysis discussed above, the compounds **1** and **2** were identified as *O,C*-diglycosides. In *O,C*-diglycosides, the terminal sugars can be released by partial acid hydrolysis. After acid hydrolysis, the degradation products of compound **1** were analyzed by LC-Q-TOF-MS and compared to an isoorientin standard (Figure 8).

It can be found from Figure 8 that the main degradation product (RT = 14.25 min) of compound **1** has the same retention time as the isoorientin standard. The main degradation product was further identified by comparing its UV spectrum, accurate mass, and retention time with those of isoorientin. By the same method, the main degradation product of compound **2** was also identified as isoorientin.

**Figure 9:**
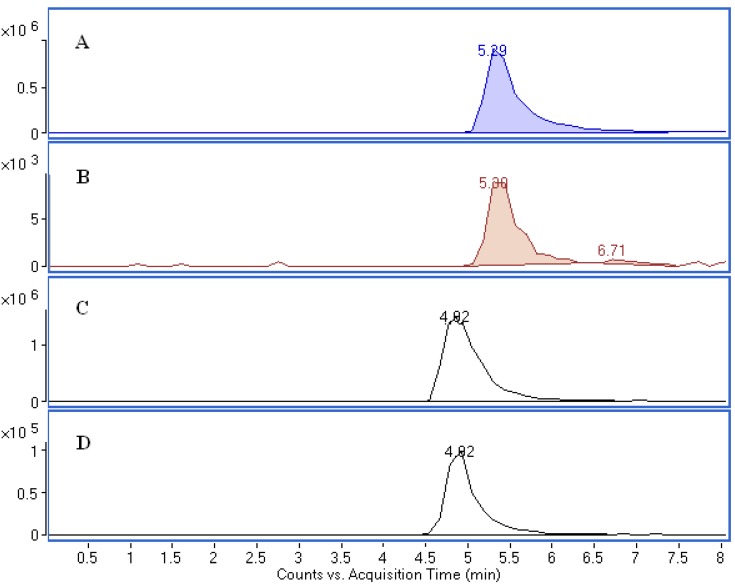
Detection of xylose and rhamnose in the degradation products by negative-mode LC/ESI-Q-TOF-MS. Extracted ion chromatograms (EIC) for xylose [M−H]^−^ 149.0455 and rhamnose [M−H]^−^ 163.0612. (**A**) xylose standard, (**B**) degradation products of compound **1**, (**C**) rhamnose standard, and (**D**) degradation products of compound **2**.

To determine whether a reducing-terminal xylose residue was present in the degradation products, we examined the degradation product solution using LC-Q-TOF-MS with a carbohydrate column. The extracted ion chromatogram (EIC) function of the TOF-MS and retention times can be used to distinguish the sugars (xylose and rhamnose) (Figure 9).

**Figure 10:**
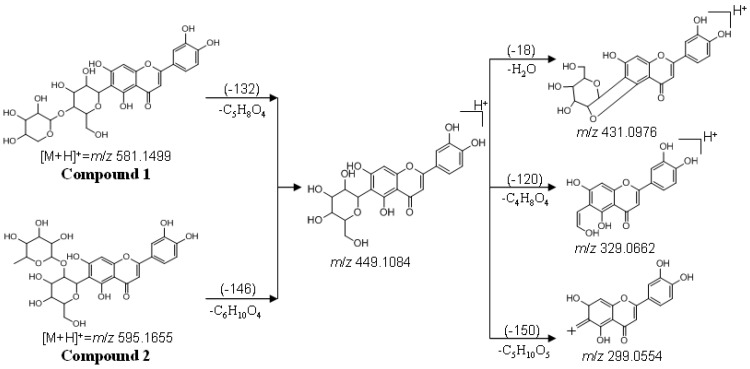
Main fragmentation pathways of compound **1** and compound **2**.

As seen from Figure 9, the extracted ion chromatograms (EIC) of the degradation products of compound **1** has the same retention time and accurate mass as those of a xylose standard. Meanwhile, the extracted ion chromatograms (EIC) of the degradation products of compound **2** has the same retention time and accurate mass as those of a rhamnose standard. These data proved that xylose and rhamnose were the reducing terminal sugars of compound **1** and compound **2**, respectively, after acid hydrolysis. Based on the combined UV, MS, and NMR data, compounds **1** and **2** were identified as isoorientin 4''-*O*-β-D-xylopyranoside (**1**), and isoorientin 2''-*O*-α-L-rhamnoside (**2**), which were also supported by their MS fragmentation pathways (Figure 10). Different types of flavonoids, including flavone *C*-glycosides, flavone *O*-glycosides, and *O,C*-diglycosides, have been identified from different bamboo species [5,19]. Of the three identified compounds in this study, isoorientin is a known antioxidant compound that has been found in most bamboo species. Compound **2** (isoorientin 2''-*O*-α-L-rhamnoside) has been found in *Sasa borealis*. To the best of our knowledge, this is the first report of the identification of compound **1** (isoorientin 4''-*O*-β-D-xylopyranoside) from the leaves of *Bambusa. textilis* McClure.

The antioxidant activity of bamboo leaves is partially due to the presence of flavonoids [20]. Flavonoids are usually extracted as mixtures from some bamboo leaves and then tested as antioxidants [21]. The proposed TLC screening method could be an easy way to evaluate the antioxidant activity of bamboo leaves or other plant extracts prior to isolation. Because of similar structures and polarity, it is also difficult to isolate and identify each antioxidant compound from bamboo leaves. 

According to known structure-activity relationships, compounds have antioxidant properties, associated with its structure. Luteolin is known as an antioxidant compound. The isolated compounds **1–3 **contain the skeletal structure of luteolin, which could be the main reason for their antioxidant activity.

## 3. Experimental

### 3.1. General

HPLC analyses were carried out on a Waters Alliance 2695 system equipped with a PDA 2996 photodiode array detector (Milford, MA, USA), using a C_18_ column (250 × 4.6 mm I.D., 5 µm, packed with R&D ODS-A, YMC, Kyoto, Japan). Isolation of active compounds was performed with a Gilson preparative HPLC GX-281/322/156 system (Gilson, Middleton, WI, USA), using a C_18_ column (250 × 20 mm I.D., 5 µm, Hydrosphere, YMC, Kyoto, Japan). MS data were recorded on an Agilent LC-Q-TOF-MS (model 6540, Agilent Technologies, Santa Clara, CA, USA). NMR spectra were recorded in DMSO-*d*_6_ (the obtained compounds dissolved in 0.5 mL solvent) on a Bruker 300 MHz spectrometer operating at 300 (^1^H) or 75 MHz (^13^C), respectively. 

### 3.2. Materials

The bamboo leaves were collected from different regions in China. The species were authenticated by Jiusheng Peng from Jiangxi Academy of Forestry and Yulong Ding from Nanjing Forestry University. Bamboo leaves were dried in the shade, ground to powder, and stored at −20 °C. The species and sampling sites of bamboo leaves are shown in Table 1.

### 3.3. Chemicals

Acetic acid, ammonium formate and 2,2-diphenyl-1-picrylhydrazyl (DPPH) were purchased from Sigma-Aldrich (St. Louis, MO, USA). The isoorientin and orientin standards were purchased from Shanghai Winherb Medical S & T Development Co., Ltd. (Shanghai, China). The standards of D-xylose and L-rhamnose hydrate were supplied by Dr. Ehrenstorfer GmbH (Augsburg, Germany). HPLC grade acetonitrile and methanol were supplied by Fisher Scientific (Fair Lawn, NJ, USA). Analytical-grade chemical was obtained from Beijing Chemical Works (Beijing, China). Ultrapure water was obtained from a Pall purification system (Purelab Plus, Pall, Port Washington, NY, USA).

### 3.4. Standard and Sample Preparation

Standard solutions of isoorientin and orientin were prepared in methanol/water (60/40, v/v) at the concentration of 100 µg/mL. Dried bamboo leaves powder (1.0 g) was mixed with 60% aqueous methanol (20 mL) and subjected to 12 h maceration with constant shaking (120 strokes/min) at room temperature [22]. The mixture was then sonicated for 30 min without heating and centrifuged at 5,000 rpm for 5 min. The supernatant was colleted and filtered through 0.45 μm nylon membrane for TLC analysis. 

### 3.5. TLC Bioautography Assay

Thin-layer chromatography (TLC) was used to separate the chemical constituents of the bamboo-leaf extracts according to our previously reported method [2]. The method employed silica gel 60 F254 precoated plates (Merck, Darmstadt, Germany) as stationary phase and mixture of ethyl acetate-formic acid-water (82:9:9, v/v/v) as mobile phase. Samples (8 μL, equivalent to a dried bamboo leaves weight of 400 µg) were were spotted in the form of bands of 8 mm width using a Camag Linomat 5 applicator (Muttenz, Switzerland). The plate was developed to a distance of 80 mm in an automatic developing chamber (ADC2, Camag). After development, the plates were immersed for 1 s in 0.05% (m/v) DPPH methanolic solution with an automatic immersion device (Camag). After immersion and removal of DPPH excess, the plates were kept in a dark box for 12 h and then photographed under visible light using a Camag Reprostar 3. The original TLC images were exported from winCATs TLC workstation (Camag) and stored as Bitmap (.bmp) files for further image processing.

### 3.6. TLC Images Processing and Evaluation of Antioxidant Activity

These .bmp files were examined with ChemPattern 2 Professional Version (a kind of fingerprint chromatography processing software, ChemMind Co. Ltd. Beijing, China). The red-green-blue (RGB) colour images were converted to grayscale intensity imagee [23]. The chromatographic peaks are shown as signals, and the integration of all peaks was done manually in triplicate. In semi-quantitative analysis, the total peak areas were used to evaluate the antioxidant activity of the extracts tested. Data were analyzed using one-way analysis of variance (ANOVA) and Duncan's multiple range test (*p* < 0.05). Results were expressed as area. Each value was the mean of triplicate measurements ± standard deviation (three measurements of each sample).

### 3.7. Preparation of Crude Extract of B. textilis McClure

Powdered leaves (100 g) of *B. textilis* McClure were extracted three times at room temperature with 1 L of 60% aqueous methanol, assisted by sonication for 30 min and maceration with constant shaking (120 strokes/min) for 12 h. After removing the solvent under reduced pressure and freeze-drying, the combined extracts were concentrated and yielded 16 g of a crude extract. The crude extract was dissolved in 100 mL water/acetonitrile (85/15, v/v) and filtered through a 0.45-μm nylon membrane.

### 3.8. HPLC Micro-fractionation

HPLC micro-fractionation of the crude solution was performed by HPLC at a flow rate of 1.0 mL/min. An HPLC mobile phase of acetonitrile/0.5% acetic acid in water (15:85 v/v) was used. Injection volume was 40 µL and detection wavelength was set at 330 nm. Collection of effluent fractions during HPLC analysis was performed on a Waters Fraction collector III (Milford, MA, USA). Fractions were collected every 1 min (1 mL) in tubes and dried under a N-EVAP-12 nitrogen evaporator (Organomation Associates, Inc., Berlin, MA, USA). Each fraction was subjected to the TLC bioautography assay.

### 3.9. Isolation of Antioxidant Compounds from *B. textilis* McClure

The isolation of antioxidant compounds was carried out using a preparative HPLC system according to our previously reported method [13]. The solvents of the preparative HPLC consisted of H_2_O (solvent A) and ACN (solvent B). The separation was carried out with elution gradient as follows: 0–3 min, 0% B; 3–7 min, 0–15% B; 7–45 min, 15% B; 45–50 min, 15–50% B; 50–52 min, 50–15% B; 52-60 min, 15% B. The flow rate was 10 mL/min with UV detection at 342 nm. Fraction F9 was purified by preparative HPLC to yield compound **1**(8 mg). Subfraction of F11 was isolated by preparative HPLC to yield compound **2** (9 mg) and compound **3** (12 mg).

### 3.10. Analytical Data

*Isoorientin 4''-O-β-D-xylopyranoside* (**1**). Yellow powder. UV λ_max_(ACN-H_2_O) 212, 270, 350 nm; HRESIMS *m*/*z* 581.1499 [M+H]^+^ (calc. for C_26_H_28_O_15_, 581.1501); ^1^H-NMR (DMSO-*d*_6_) δ: 6.57 (1H, s, H-3), 6.36 (1H, s, H-8), 7.22 (1H, s, H-2'), 6.77 (1H, d, H-5'), 7.29 (1H, d, H-6'), 4.32 (1H, d, H-1''), 4.55 (1H, d, H-1'''), 3.00–4.50 (11H, m, Sugar); ^13^C-NMR (DMSO-*d*_6_) δ: 161.9 (C-2), 103.2 (C-3), 182.3 (C-4), 161.8 (C-5), 108.5 (C-6), 164.1 (C-7), 93.9 (C-8), 156.8 (C-9), 103.7 (C-10), 121.9 (C-1'), 113.7 (C-2'), 146.2 (C-3’), 150.2 (C-4'), 116.5 (C-5'), 119.4 (C-6'), 74.6 (C-1''), 71.7 (C-2''), 74.1 (C-3''), 81.4 (C-4''), 82.0 (C-5''), 61.9 (C-6''), 106.5 (C-1'''), 76.6 (C-2''), 78.8 (C-3'''), 70.8 (C-4'''), 66.1 (C-5'''). Copies of the ^1^H- and ^13^C-NMR spectra are available in the Supplementary Material.

*Isoorientin 2''-O-α-L-rhamnoside* (**2**). Yellow powder. UV λ_max_(ACN-H_2_O) 212, 270, 350 nm; HRESIMS *m*/*z* 595.1655 [M+H]^+^ (calc. for C_27_H_30_O_15_, 595.1657); ^1^H-NMR (DMSO-*d*_6_) δ: 6.55 (1H, s, H-3), 6.36 (1H, s, H-8), 7.28 (1H, s, H-2'), 6.78 (1H, d, H-5'), 7.29 (1H, d, H-6'), 4.27 (1H, d, H-1''), 4.97 (1H, d, H-1'''), 0.49 (3H, d, H-6'''), 3.00–4.50 (10H, m, Sugar); ^13^C-NMR (DMSO-*d*_6_) δ: 163.0 (C-2), 103.1 (C-3), 182.5 (C-4), 161.7 (C-5), 109.4 (C-6), 163.9 (C-7), 93.2 (C-8), 156.6 (C-9), 104.2 (C-10), 121.8 (C-1'), 113.6 (C-2'), 146.2 (C-3'), 150.2 (C-4'), 116.5 (C-5'), 119.4 (C-6'), 74.9 (C-1''), 80.5 (C-2''), 76.1 (C-3''), 71.0 (C-4''), 81.9 (C-5''), 62.2 (C-6''), 100.8 (C-1'''), 75.0 (C-2'''), 70.8 (C-3'''), 71.4 (C-4'''), 68.7 (C-5'''), 18.0 (C-6'''). Copies of the ^1^H- and ^13^C-NMR spectra are available in the Supplementary Material.

### 3.11. LC-Q-TOF-MS Analysis

Analysis of flavonoids: the qualitative analysis of the isolated compounds was performed using LC-Q-TOF-MS equipped with a jet stream ESI interface. Chromatographic separation was carried out on a C_18_ column (250 × 4.6 mm I.D., 5 µm, packed with R&D ODS-A, YMC, Kyoto, Japan) at 30 °C. The mobile phase consisted of phase A (aqueous 0.1% acetic acid) and phase B (acetonitrile) in the ratio 85/15 (v/v). The flow rate was 1.0 mL/min and the T splitter gave a flow rate of about 0.20 mL/min toward the MS detector. The TOF/MS system was operated in positive mode and the operating parameters were as follows: drying gas (N_2_) flow rate, 10 L/min; drying gas temperature, 350 °C; Nebulizer, 60 psi; capillary voltage, 3,500 V; skimmer voltage, 65 V; fragmentor voltage, 175 V; sheath gas temperature, 350 °C; nozzle voltage, 500 V. Mass spectra were acquired in the *m/z* range of 20 to 1500. The MS data were collected in an auto MS/MS mode using a ramped collision energy setting with slope 5 (collision Energy = slope × *m/z* / 100). Two reference masses were used: 149.0233 and 922.0098. During the analysis, the reference masses were infused to the MS system to allow constant mass correction. The elemental composition of the selected peaks were obtained by the accurate mass of the precursor and product ions. All the operations and analysis of data were controlled under MassHunter B.04 software.

Analysis of sugars: the qualitative analysis of the sugars was performed using LC-Q-TOF-MS equipped with a jet stream ESI interface. Chromatographic separation was carried out on a carbohydrate column (300 × 3.9 mm I.D., Part No WAT084038, Waters, Ireland) at 30 °C. The mobile phase consisted of phase A (aqueous 5 mM ammonium formate) and phase B (acetonitrile) in the ratio 25/75 (v/v). The flow rate was 1.0 mL/min and the T splitter gave a flow rate of about 0.20 mL/min toward the MS detector. 

The TOF/MS system was operated in negative mode and the main operating parameters were as follows: drying gas (N2) flow rate, 8 L/min; drying gas temperature, 350 °C; Nebulizer, 40 psi; capillary voltage, 3500 V; skimmer voltage, 65 V; fragmentor voltage, 60 V; sheath gas temperature, 400 °C; nozzle voltage, 1500 V. The MS data were collected in an auto MS/MS mode at a fixed collision energy of 5 eV.

### 3.12. Acid Hydrolysis of Compounds ***1*** and ***2***

A solution of compound **1** (2.5 mg) in MeOH-H_2_O (3:2, v/v, 5 mL) and 2 M aqueous HCl (5 mL) was refluxed for 1.5 h in a water bath (85 °C). After repeated evaporation of the aqueous solution by adding MeOH to reduce the acid, the reaction mixture was concentrated to 2 mL under reduced pressure [6,24]. A aliquot of sample (0.5 mL of the reaction mixture and 0.5 mL MeOH) was filtered through a 0.22-μm nylon membrane for LC-Q-TOF-MS analysis. Sugars were identified by LC-Q-TOF-MS and compared to authentic sugars. By the same method, compound **2 **was also analyzed. 

## 4. Conclusions

In summary, a combination strategy was provided for the screening and identification of antioxidative compounds of bamboo leaves. A TLC bioautography method combined with an image processing method could be an easy way to screen bamboo extracts for antioxidant activity. Three antioxidant compounds, including isoorientin 4"-*O*-β-D-xylopyranoside (**1**), isoorientin 2"-*O*-α-L-rhamnoside (**2**) and isoorientin (**3**) were identified by their UV, MS, and NMR spectra. The leaf extract of *B. textilis* McClure shows promise as a new source of natural antioxidants.

## Supplementary Materials

Supplementary materials can be accessed at: http://www.mdpi.com/1420-3049/17/10/12297/s1.

## References

[1] Kweon M.H., Hwang H.J., Sung H.C. (2001). Identification and antioxidant activity of novel chlorogenic acid derivatives from bamboo (*Phyllostachys. edulis*). J. Agric. Food Chem..

[2] Wang J., Yue Y.D., Jiang H., Tang F. (2012). Rapid screening for flavone *C*-glycosides in the leaves of different species of bamboo and simultaneous quantitation of four marker compounds by HPLC-UV/DAD. Int. J. Anal. Chem..

[3] Hoyweghen L.V., Karalic I., Calenbergh S.V., Deforce D., Heyerick A. (2010). Antioxidant flavone glycosides from the leaves of *Fargesia. robusta*. J. Nat. Prod..

[4] Zhang Y., Bao B., Lu B., Ren Y., Tie X., Zhang Y. (2005). Determination of flavone *C*-glucosides in antioxidant of bamboo leaves (AOB) fortified foods by reversed-phase high-performance liquid chromatography with ultraviolet diode array detection. J. Chromatogr. A.

[5] Zhang Y., Jiao J., Liu C., Wu X., Zhang Y. (2008). Isolation and purification of four flavone *C*-glycosides from antioxidant of bamboo leaves by macroporous resin column chromatography and preparative high-performance liquid chromatography. Food Chem..

[6] Park H.S., Lim J.H., Kim H.J., Choi H.J., Lee I.S. (2007). Antioxidant flavone glycosides from the leaves of *Sasa. borealis*. Arch. Pharm. Res..

[7] Jasprica I., Bojic M., Mornar A., Besic E., Bucan K., Medic-Saric M. (2007). Evaluation of antioxidative activity of croatian propolis samples using DPPH and ABTS ^+^ stable free radical assays. Molecules.

[8] Zhao J., Zhang J.S., Yang B., Lv G.P., Li S.P. (2010). Free radical scavenging activity and characterization of sesquiterpenoids in four species of curcuma using a TLC bioautography assay and GC-MS analysis. Molecules.

[9] Kusznierewicz B., Piekarska A., Mrugalska B., Konieczka P., Namieśnik J., Bartoszek A. (2012). Phenolic composition and antioxidant properties of polish blue-berried honeysuckle genotypes by HPLC-DAD-MS, HPLC postcolumn derivatization with ABTS or FC, and TLC with DPPH visualization. J. Agric. Food Chem..

[10] Olech M., Komsta Ł., Nowak R., Cieśla Ł., Waksmundzka-Hajnos M. (2012). Investigation of antiradical activity of plant material by thin-layer chromatography with image processing. Food Chem..

[11] Cimpoiu D.C.  (2006). Analysis of some natural antioxidants by thin-layer chromatography and high performance thin-layer chromatography. J. Liq. Chromatogr. R. T..

[12] Badarinath A.V., Mallikarjuna K., Madhau Sudhana Chetty C., Ramkanth S., Rajan T.V.S., Guanaprahash K. (2010). A review on *in vitro* antioxidant methods: comparisions, correlations and considerations. Int. J. Pharm. Tech. Res..

[13] Wang J., Yue Y.D., Tang F., Sun J. (2012). Screening and analysis of the potential bioactive components in rabbit plasma after oral administration of hot-water extracts from leaves of *Bambusa. textilis* McClure. Molecules.

[14] Ruiz-Terán F., Medrano-Martínez A., Navarro-Ocaña A. (2010). Antioxidant and free radical scavenging activities of plant extracts used in traditional medicine in Mexico. Afr. J. Biotechnol..

[15] Rumzhum N.N., Rahman M.M., Kazal M.K. (2012). Antioxidant and cytotoxic potential of methanol extract of *Tabernaemontana. divaricata* leaves. Int. Curr. Pharm. J..

[16] Lapornik B., Wondra A.G., Prošek M. (2004). Comparison of TLC and spectrophotometric methods for evaluation of the antioxidant activity of grape and berry anthocyanins. J. Planar Chromatogr..

[17] Ignatov S., Shishniashvili D., Ge B., Scheller F.W., Lisdat F. (2002). Amperometric biosensor based on a functionalized gold electrode for the detection of antioxidants. Biosens. Bioelectron..

[18] Figueirinha A., Paranhos A., Pérez-Alonso J.J., Santos-Buelga C., Batista M.T. (2008). Cymbopogon citratus leaves: characterization of flavonoids by HPLC-PDA-ESI/MS/MS and an approach to their potential as a source of bioactive polyphenols. Food Chem..

[19] Lv Z., Dong J., Zhang B. (2011). Rapid identification and detection of flavonoids compounds from bamboo leaves by LC-(ESI)-IT-TOF/MS. BioResources.

[20] Hasegawa T., Tanaka A., Hosoda A., Takano F., Ohta T. (2008). Antioxidant *C*-glycosyl flavones from the leaves of *Sasa. kurilensis* var. *gigantea*. Phytochemistry.

[21] Mu J., Uehara T., Li J., Furuno T. (2004). Identification and evaluation of antioxidant activities of bamboo extracts. For. Stud. China.

[22] Zeraik M.L., Yariwake J.H. (2010). Quantification of isoorientin and total flavonoids in *Passiflora. edulis* fruit pulp by HPLC-UV/DAD. Microchem. J..

[23] Tian R.T., Xie P.S., Liu H.P. (2009). Evaluation of traditional Chinese herbal medicine: Chaihu (Bupleuri radix) by both high-performance liquid chromatographic and high-performance thin-layer chromatographic fingerprint and chemometric analysis. J. Chromatogr. A.

[24] Ragab E.A., Hosny M., Kadry H.A., Ammar H.A. (2010). Flavanone glycosides from *Gleditsia. caspia*. J. Nat. Prod..

