# Supplementary material for: TLC Screening for Antioxidant Activity of Extracts from Fifteen Bamboo Species and Identification of Antioxidant Flavone Glycosides from Leaves of Bambusa. textilis McClure
Source: Molecules. 2012 Oct 19;17(10):12297–311. doi: 10.3390/molecules171012297 (PMC6268801; doi:10.3390/molecules171012297)

Supplementary Materials

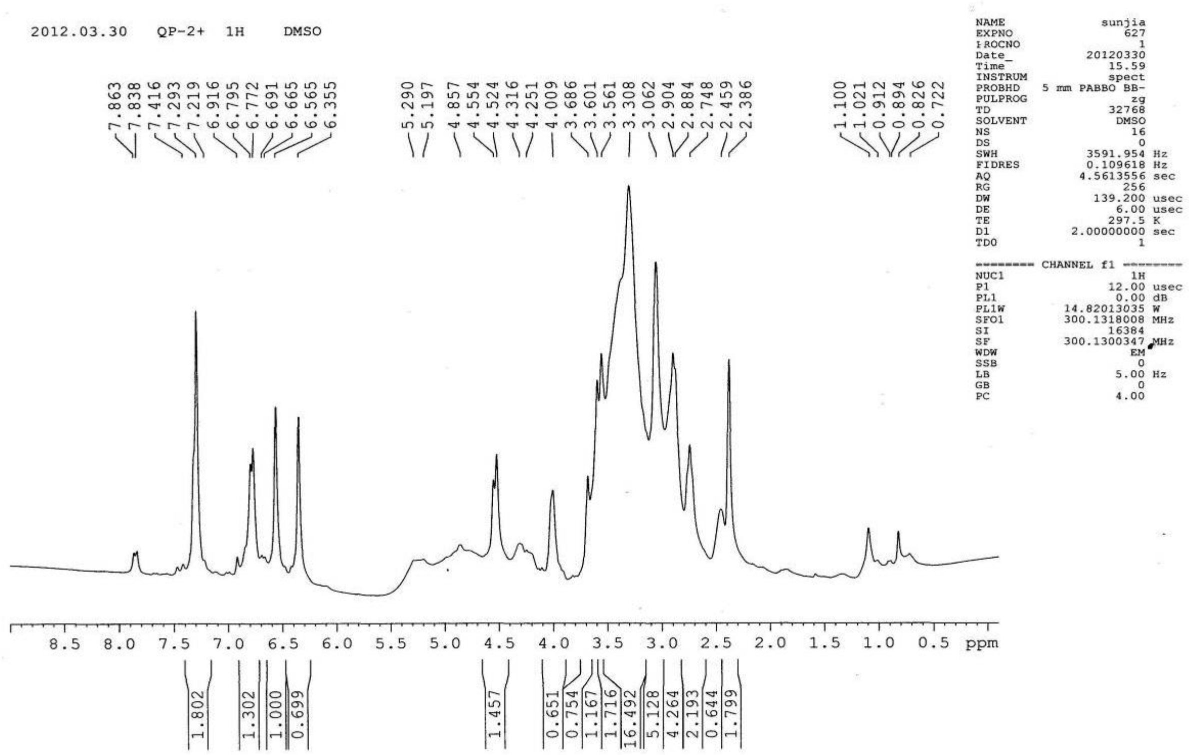

Figure S1. ^{1}H-NMR of compound **1**.

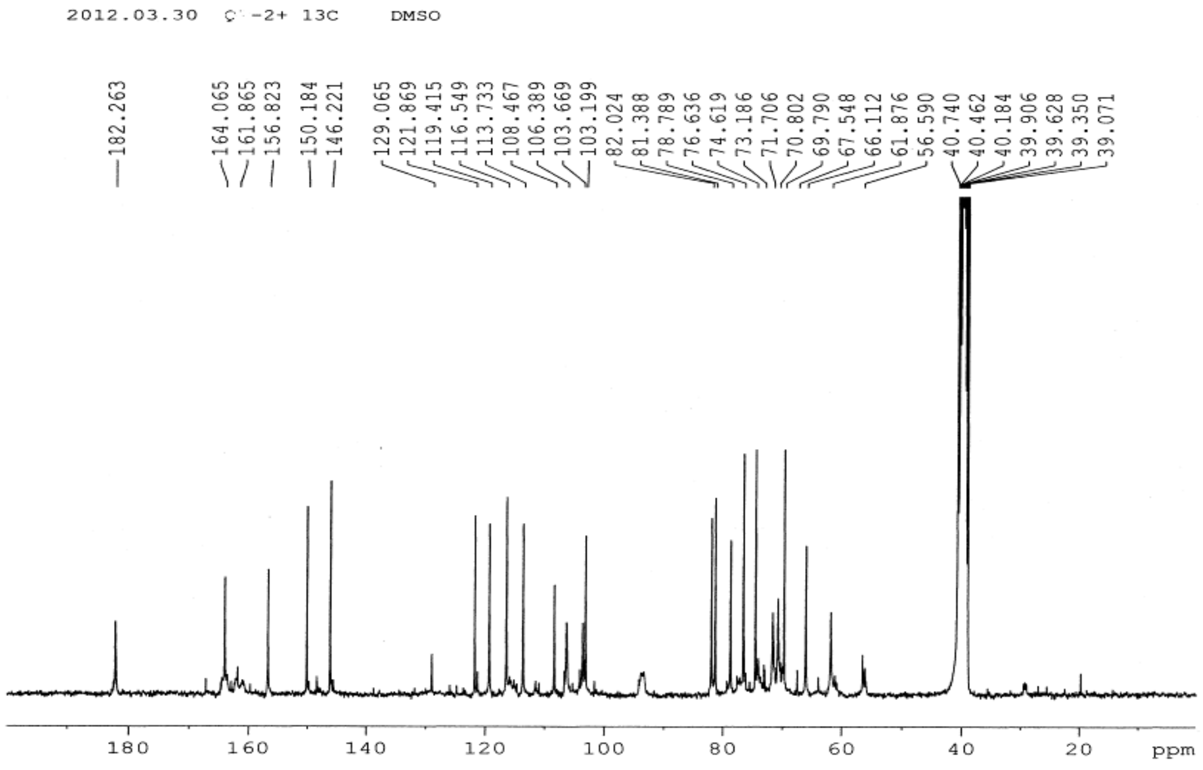

Figure S2. ^{13}C-NMR of compound **1**.

Figure S3. DEPT 135 NMR spectrum for compound **1**.

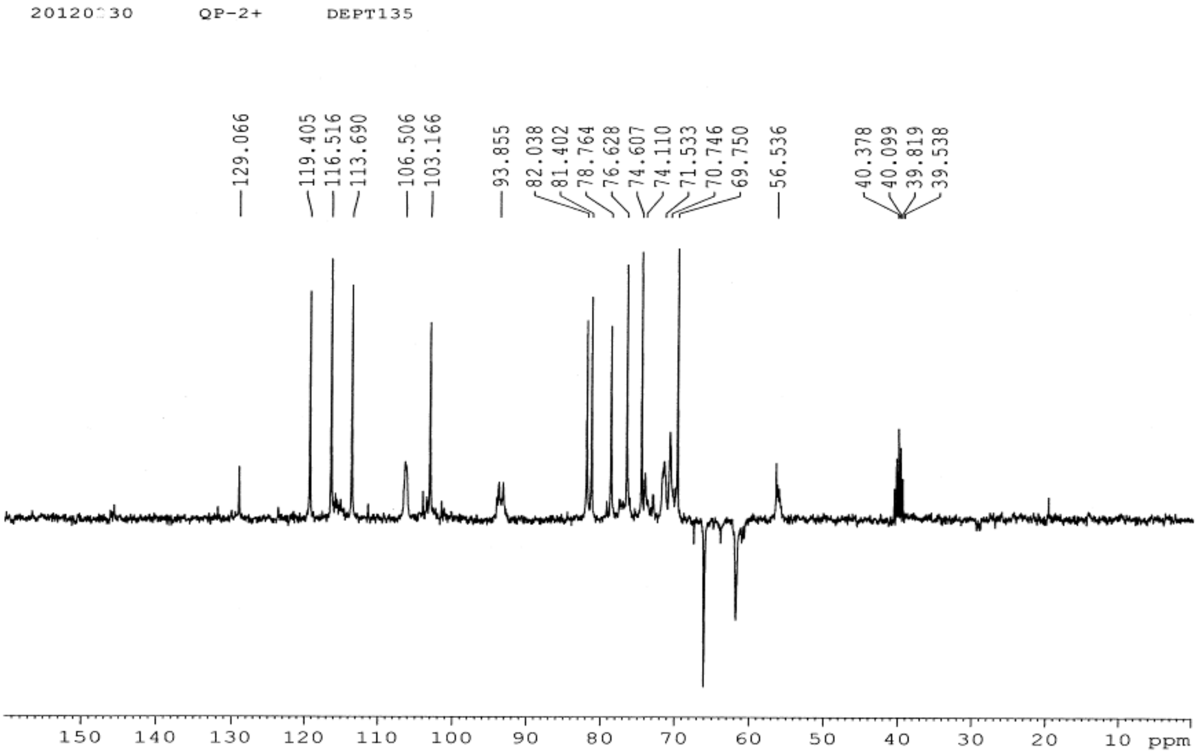

Figure S4. DEPT 90 NMR spectrum for compound **1**.

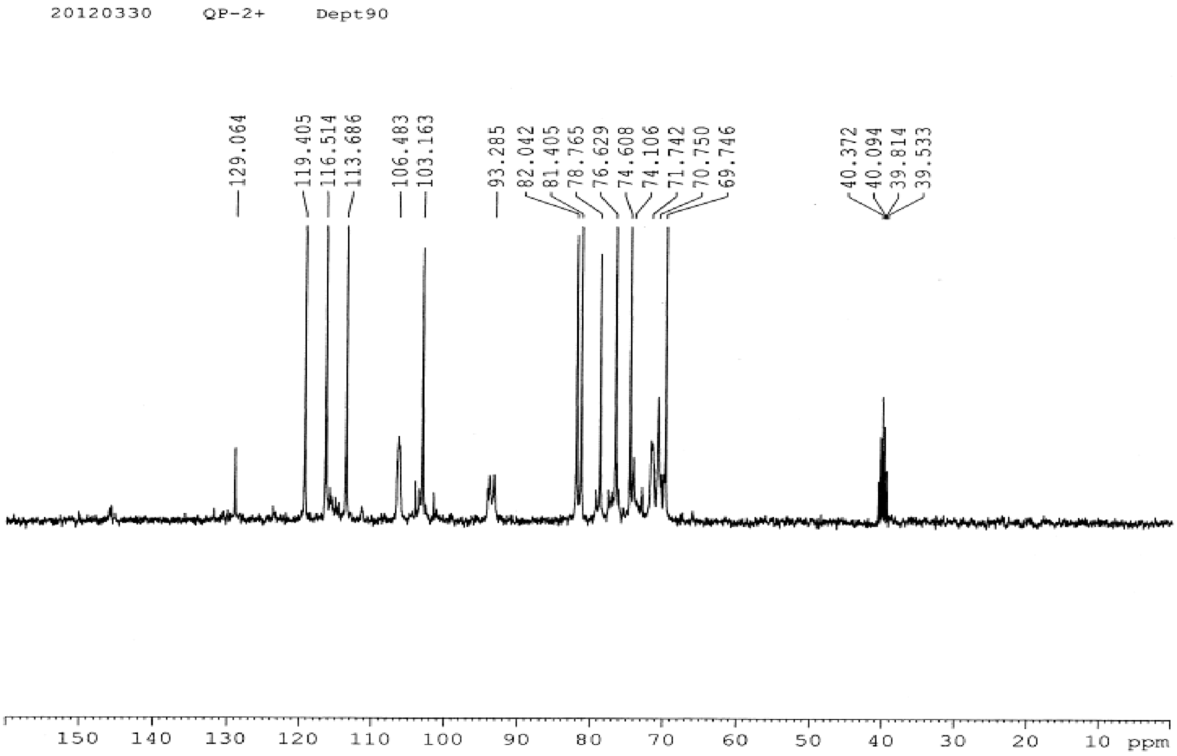

Figure S5. ^{1}H-NMR of compound **2**.

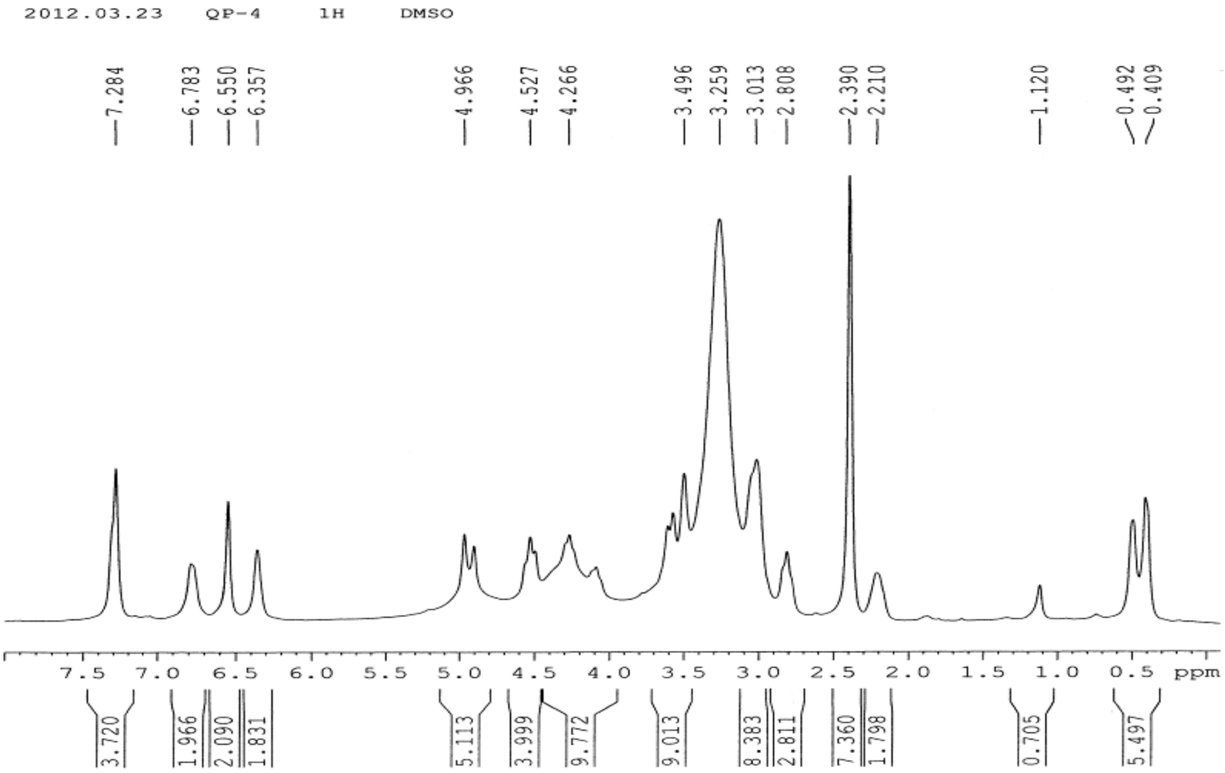

Figure S6. ^{13}C-NMR of compound **2**.

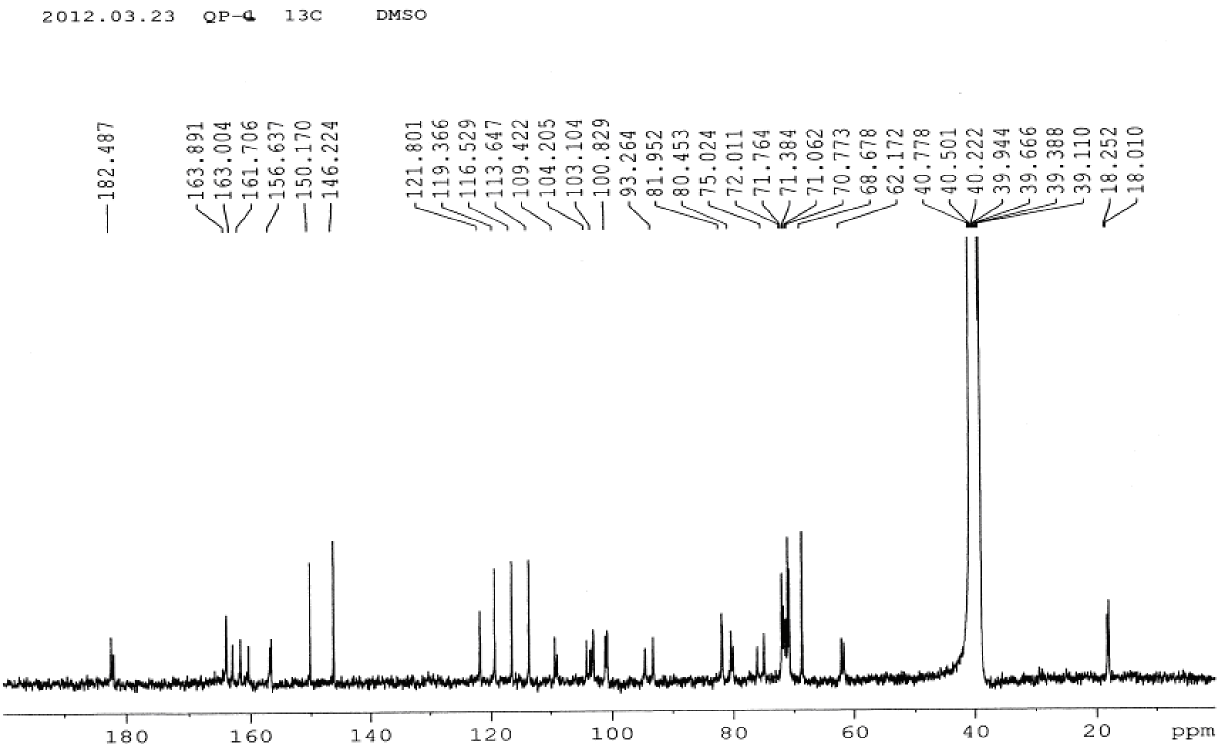

Figure S7. DEPT 135 NMR spectrum for compound **2**.

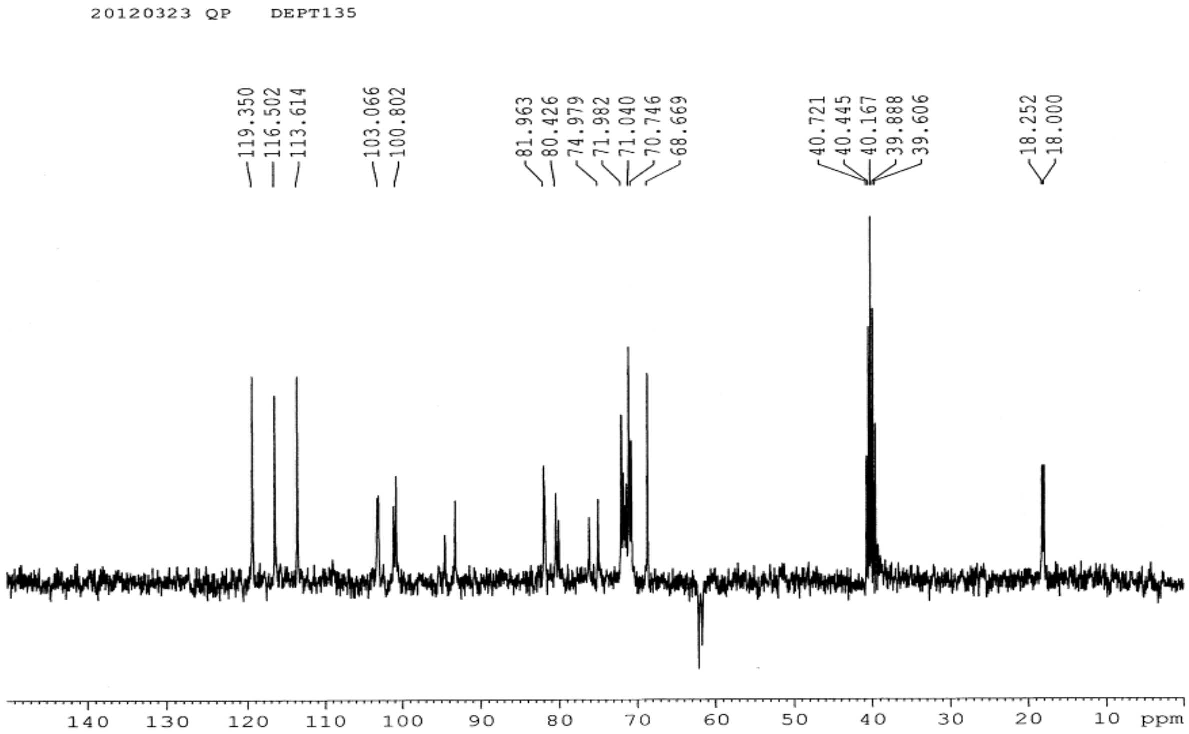

Figure S8. DEPT 90 NMR spectrum for compound **2**.

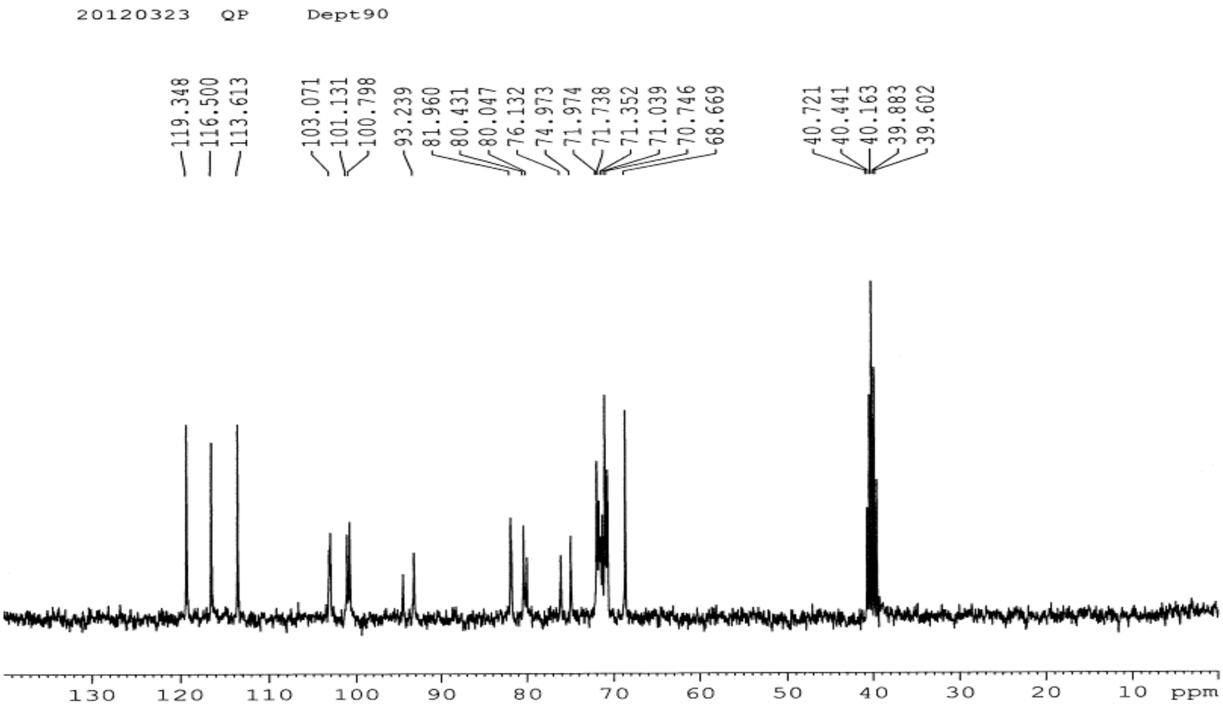

Supplement: Supplementary file 1 [file molecules-17-12297-s001.pdf]
